# A Consideration of the Methods for Demonstrating Bacilli in the Urine

**Published:** 1919

**Authors:** 


**A Consideration of the Methods for Demonstrating Bacilli in the Urine.** By E. M. Watson. *The American Journal of the Medical Sciences*, November, 1918.

The means used for the demonstrating of tubercle bacilli in the urine have until recently been the simple settling of the sediment by natural gravitation over a period of hours, or by centrifugalization of the same. From this sediment a few loopfuls of material were smeared on a glass slide, fixed in the flame, and stained either by Ehrlich's anilin-water-gentian-violet method or by the carbol-fuchsin solution of Ziehl.

The author gives in detail the following methods :

(1) Walker's,
(2) Uhlenhuth's,
(3) Ellerman and Erlandsen's,
(4) Lange and Nitsche's,
(5) Kozlow's,
(6) Petroff's,
(7) Crabtree's, and finally the procedure described below, which has been found excellent.

(8) *Method. a)* Irrigate the glans penis and urethra with sterile water.

*b*) An amount of 200 cc. of urine is collected into 3 glasses, the last of which is a conical-shaped sedimenting glass of 250 cc. capacity, fitting in to an ordinary high-powered electric laboratory centrifuge.

*c*) The specimen (about 200 cc.) is placed in the electric centrifuge and revolved for 5 minutes at moderate speed.

*d*) The entire specimen is subjected to another centrifugalization at high speed for 30 to 40 minutes.

*e*) The supernatent urine is decanted and the sediment used for preparing 3 glass slides.

*f*) The slides are allowed to dry, and are fixed in the Bunsen flame.

*g*) If smears are thick they are placed in 5 o/o acid (HCl) alcohol for 2 minutes, and again fixed in the Bunsen flame.

*h*) The slides are stained in Ziehl's solution for 10 minutes, washed in running water, decolorized, and counterstained in Loeffler's methylene blue.

If the attempts to demonstrate the offending organisms in cases of suspected urogenital tuberculosis have yielded no results, resort may be had to animal inoculation which has, in recent years, come into general use. The guinea-pig test, however, is not infallible, and occasionally the organisms may be demonstrated by centrifugalization and staining when the guinea-pig test is negative.

